# Deep Learning-Based Differentiation between Mucinous Cystic Neoplasm and Serous Cystic Neoplasm in the Pancreas Using Endoscopic Ultrasonography

**DOI:** 10.3390/diagnostics11061052

**Published:** 2021-06-08

**Authors:** Leang Sim Nguon, Kangwon Seo, Jung-Hyun Lim, Tae-Jun Song, Sung-Hyun Cho, Jin-Seok Park, Suhyun Park

**Affiliations:** 1 School of Electrical and Electronics Engineering, Chung-Ang University, Seoul 06974, Korea; leangsim.arcs@gmail.com (L.S.N.); skangwon@naver.com (K.S.); 2 Division of Gastroenterology, Department of Internal Medicine, Inha University School of Medicine, Incheon 22332, Korea; limren@naver.com; 3 Department of Gastroenterology, Asan Medical Center, University of Ulsan College of Medicine, Seoul 05505, Korea; medi01@naver.com (T.-J.S.); doctorcho5@hanmail.net (S.-H.C.); 4 Department of Electronic and Electrical Engineering, Ewha Womans University, Seoul 03760, Korea

**Keywords:** endoscopic ultrasonography, mucinous cystic neoplasm, serous cystic neoplasm, deep learning, convolutional neural network, pancreatic cystic neoplasms, diagnostic imaging

## Abstract

Mucinous cystic neoplasms (MCN) and serous cystic neoplasms (SCN) account for a large portion of solitary pancreatic cystic neoplasms (PCN). In this study we implemented a convolutional neural network (CNN) model using ResNet50 to differentiate between MCN and SCN. The training data were collected retrospectively from 59 MCN and 49 SCN patients from two different hospitals. Data augmentation was used to enhance the size and quality of training datasets. Fine-tuning training approaches were utilized by adopting the pre-trained model from transfer learning while training selected layers. Testing of the network was conducted by varying the endoscopic ultrasonography (EUS) image sizes and positions to evaluate the network performance for differentiation. The proposed network model achieved up to 82.75% accuracy and a 0.88 (95% CI: 0.817–0.930) area under curve (AUC) score. The performance of the implemented deep learning networks in decision-making using only EUS images is comparable to that of traditional manual decision-making using EUS images along with supporting clinical information. Gradient-weighted class activation mapping (Grad-CAM) confirmed that the network model learned the features from the cyst region accurately. This study proves the feasibility of diagnosing MCN and SCN using a deep learning network model. Further improvement using more datasets is needed.

## 1. Introduction

Endoscopic ultrasonography (EUS) is used for diagnosing pancreatic cystic neoplasms (PCN) such as intraductal papillary mucinous neoplasm (IPMN), mucinous cystic neoplasm (MCN), serous cystic neoplasm (SCN), and other cystic cases [1,2,3,4,5,6,7]. As the spectrum of PCN varies widely from benign to malignant, the accurate diagnosis of PCN is required. While MCN has the potential to develop into a malignancy needing early surgical resection, SCN involves a relatively small risk of malignancy [1,2,5,7,8,9,10,11,12,13]. Thus, it is critical to determine whether a cyst is MCN or SCN [1,9,13,14], which can be categorized as mucinous and non-mucinous cysts, respectively [1,6,7,10].

The diagnosis of MCN and SCN is challenging because of the lack of criteria for differentiation [1,2,6,10,12,14,15]. Nagashio et al. [6] utilized fluid markers such as the carcinoembryonic antigens (CEA), carbohydrate antigens (CA 19-9 and CA 125), amylase, as well as cytology to classify different pancreatic lesions and to differentiate between malignant and benign lesions. The study showed that the combination of fluid markers is useful for differentiation between mucinous and non-mucinous lesions with an accuracy of 77.8%. Gaddam et al. [7] also tried to find the optimal cutoff value for the CEA level to diagnose between mucinous and non-mucinous cystic lesions, and the result from the study could reach an AUC around 0.77. Pérez et al. [5] investigated the impact of molecular analysis for PCN classification by analyzing the combination of morphological, cytological, and biochemical analysis to differentiate between mucinous and non-mucinous PCNs. Their study showed that molecular analysis was effective for the differentiation of mucinous cysts from non-mucinous cysts. Wang Y. et al. [9] classified MCN and SCN by investigating the alterations of glycopatterns in cystic fluids and demonstrated that the variation in glycopatterns and glycoproteins are associated with the differences between MCN and SCN. Biomarkers can be good indicators in the diagnosis but have limitations if used alone.

Various imaging modalities such as endoscopic ultrasound (EUS), magnetic resonance imaging (MRI), computed tomography (CT), and positron emission tomography (PET) are used for visual diagnosis of MCN and SCN [1,3,4,5,7,10,12]. Wang G. et al. [13] proposed a method to differentiate PCNs by combining the observations from PET/CT with multiparametric analysis such as the maximum and mean of standardized uptake values, metabolic tumor volume (MTV), and total lesion glycolysis (TLG). Their study achieved an AUC up to 0.810. Diagnosis using imaging modalities mainly relies on visual observations and biopsies. Among the imaging modalities, EUS has the advantage of capturing internal structures [1,4,12,16] and can be combined with a biopsy by using fine-needle aspiration (FNA) [3,10]. Köker et al. [17] performed the EUS-FNA to investigate the effectiveness of CEA level for differentiating low risk IPMN from low risk MCN. The study achieved an AUC around 0.930 for differentiating between low risk IPMN and low risk MCN. Zhang et al. [1] suggested certain criteria based on the visual features of pancreatic cysts in EUS images such as honeycomb cysts, latticed cysts, and round shaped cysts featuring nodules and calcification. In addition to the visual information, the carcinoembryonic antigen (CEA) level analyzed from cyst fluid was utilized. These criteria helped achieve 82.93% accuracy. Kubo et al. [2] also reported features to diagnose PCN in the EUS images, which were microcystic areas, mural nodules, the number of cystic formations, locularity, and the cystic component. Despite providing pathological information for the diagnosis of cysts, EUS with FNA has some safety concerns as it can lead to severe outcomes for non-mucinous cases, such as peritoneal dissemination or gastric seeding due to the leakage of cyst fluid [18,19].

Using deep learning techniques such as convolutional neural networks (CNNs), complicated features that are unrecognizable to human vision can be extracted. Thus, deep learning technology is widely used in classification tasks in the medical imaging field [14,15,16,20,21,22]. Kurita et al. [15] investigated the feasibility of neural network models for differentiating between malignant and benign pancreatic lesions by utilizing the carcinoembryonic antigen (CEA), cytology, and other clinical information, and showed an accuracy of 92.9%. Kuwahara et al. [20] used the CNN model to diagnose benign and malignant cases of IPMN by utilizing EUS images of PCN lesions, and achieved 86.2% accuracy.

The recent studies have proven that the deep learning techniques can be applied to diagnosis of pancreatic disease. Thus far, to the best of our knowledge, no study has been conducted on the classification of MCN and SCN from EUS images using CNN models. In this study, we aim to implement CNN models to differentiate between MCN and SCN from EUS images of the pancreas. The performance of the CNN model for this classification is thoroughly evaluated.

## 2. Materials and Methods

### 2.1. EUS Data

#### 2.1.1. Data Acquisition

All procedures were performed between December 2010 and December 2020 at the endoscopic center of Inha University Hospital and Asan Medical Center. An ultrasound scanning system (SSD 5500, 5 and 10; Aloka, Tokyo, Japan) with linear (GF-UCT 240; Olympus Optical corp. Ltd., Tokyo, Japan) and radial (GF-UE 260; Olympus Optical corp. Ltd., Tokyo, Japan) echoendoscopes was used. The examination was conducted by endosonographers with at least five years of experience. Endosonographers at two separate sites independently acquired and completed the analysis of the EUS images. The clinical data were analyzed for image findings and pathological diagnosis of the patients. To validate the diagnosis of unclear cases, FNA or histology analysis through surgery was performed and 44% of the patients experienced this additional level of diagnosis. This study was reviewed and approved by the Institutional Review Boards of Inha University Hospital (2020-05-002) and of Asan Medical Center (2020-1290-0001).

**Figure 1:**
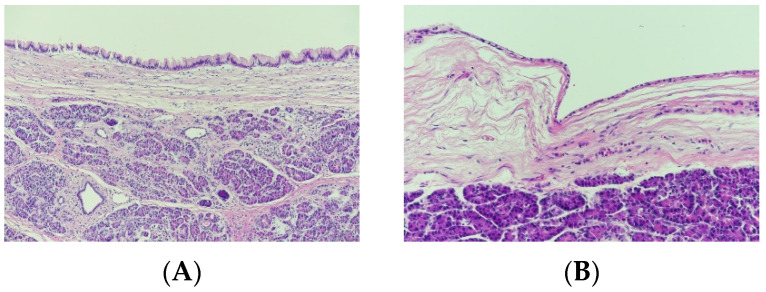
Histology images (200×) for (**A**) mucinous cystic neoplasm and (**B**) serous cystic neoplasm cases.

The EUS data were acquired from 47 MCN and 31 SCN patients at the 1st hospital and 13 MCN and 18 SCN patients at the 2nd hospital. The characteristics of the patients and lesions for this study are summarized in Table 1. Figure 1 shows a sample of histology images for MCN (Figure 1A) and SCN (Figure 1B) cases.

#### 2.1.2. Data Preparation and Preprocessing

**Figure 2:**
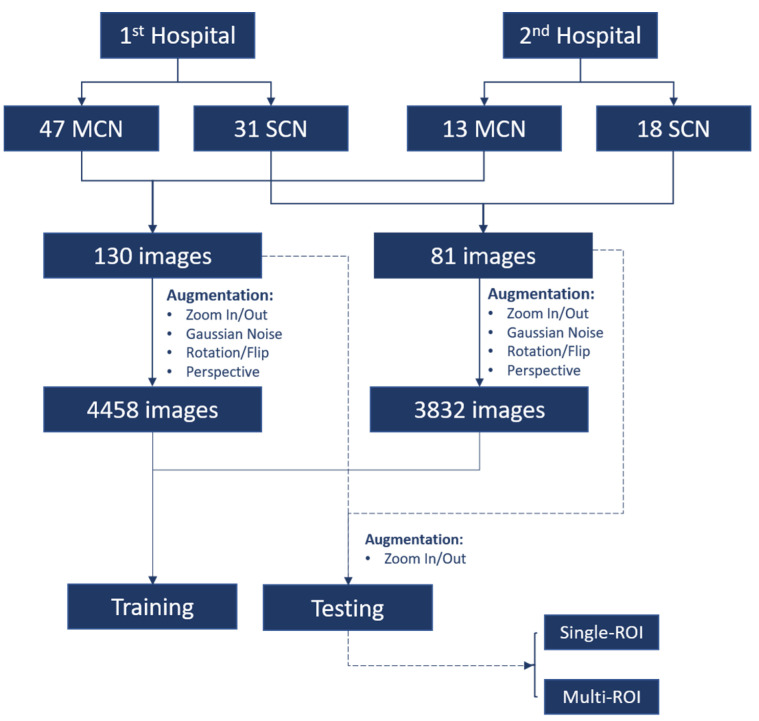
Diagram of data preparation and preprocessing. There were 109 patients, including 60 mucinous cystic neoplasm (MCN) patients and 49 serous cystic neoplasm (SCN) patients providing 130 and 81 endoscopic ultrasonography images for MCN and SCN, respectively.

**Figure 3:**
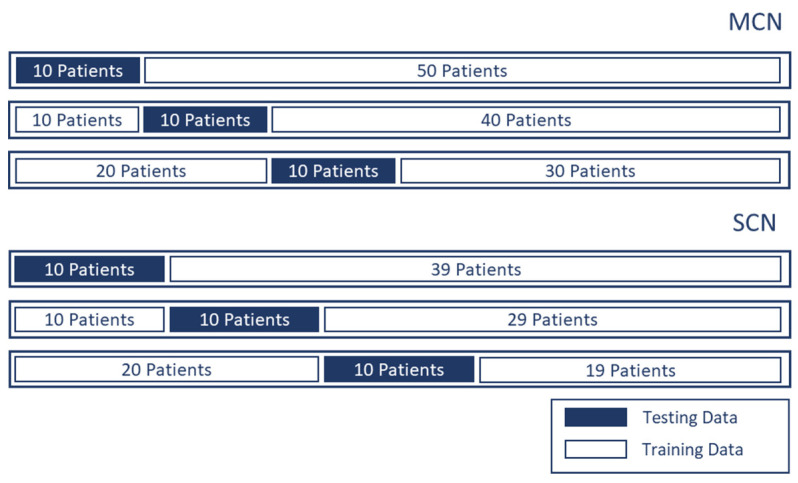
Three groups of holdout validation. Ten patients were selected from each class for testing and the rest were used to provide training data.

Figure 2 illustrates the data preparation and preprocessing procedure for this study. From each patient’s data (out of 109 patients), 1–4 EUS images were extracted. For MCN and SCN cases, 130 and 81 images, respectively, were selected. The region surrounding the cysts in the EUS image was selected as the region of interest (ROI). Unnecessary regions including the metadata information and the circular hollow region (radial EUS probe) or semicircular hollow region (linear EUS probe) were excluded from the ROI. For the test data of the CNN model, two types of ROI selections were made to check whether the performance of the network model was affected by the ROI selection. The first type of ROI was selected in an aspect ratio of 1:1 (i.e., a square); this type of ROI selection is denoted as single-ROI. Another type of ROI selection involved varying the size and aspect ratio of four different ROIs; this type of ROI selection is denoted by multi-ROI. In order to validate the proposed model without bias, we performed the hold out validation three times. From each group, 10 different patients were selected for each class (i.e., MCN and SCN) while the rest of the data were used as training data, as shown in Figure 3.

#### 2.1.3. Data Augmentation for Training Data

Data augmentation was used to increase the amount of training data and further help the generalization of the network decision. The augmentation techniques used in this study are flip, rotation, translation, zoom in, zoom out, adding Gaussian noise, and perspective transformation [23]. Perspective transformation was utilized to simulate the deformation of the cyst in the EUS image.

**Figure 4:**
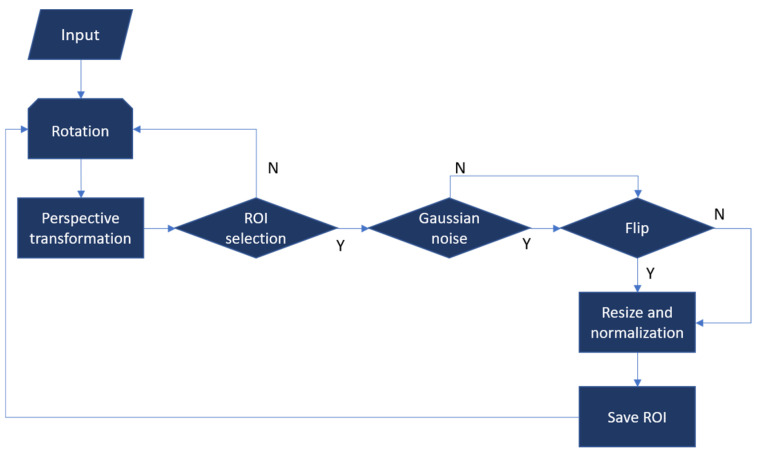
Data augmentation process for training data. Y and N represent the decision as to whether ROI can be selected, noise is added, and flipping is applied.

**Figure 5:**
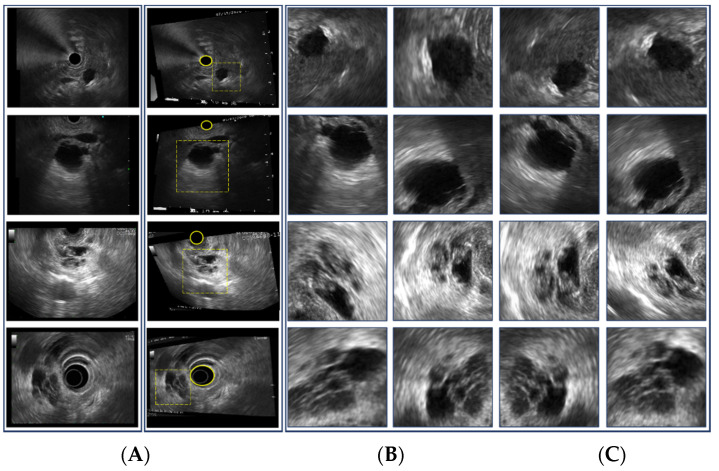
Examples of data augmentation for training data. (**A**) Original EUS image, (**B**) image after perspective transformation, (**C**) selected ROIs. The yellow circle in (**B**) represents the circular or semicircular hollow region owing to the EUS probe, while the dotted square in (**B**) represents examples of ROI selection.

Data augmentation for the training data is illustrated in Figure 4. Examples of the augmentation process are shown in Figure 5. The augmentation process starts from a grayscale EUS image and is followed by rotation and random perspective transformation. Then, ROIs in arbitrary rectangular shapes were selected manually as shown in Figure 5B. Selected ROIs were flipped after Gaussian noise was added. Finally, each ROI was resized to act as the input of the CNN model (i.e., 244 × 244 in this study). Intensities were normalized in the range of (0, 1). The examples of the augmented ROIs are shown in Figure 5C. Given that the EUS image was in grayscale, the grayscale images were repeated for the three-color channels of the input for the CNN model.

### 2.2. Deep Learning Approach

#### 2.2.1. Deep Learning Network Architecture

**Figure 6:**
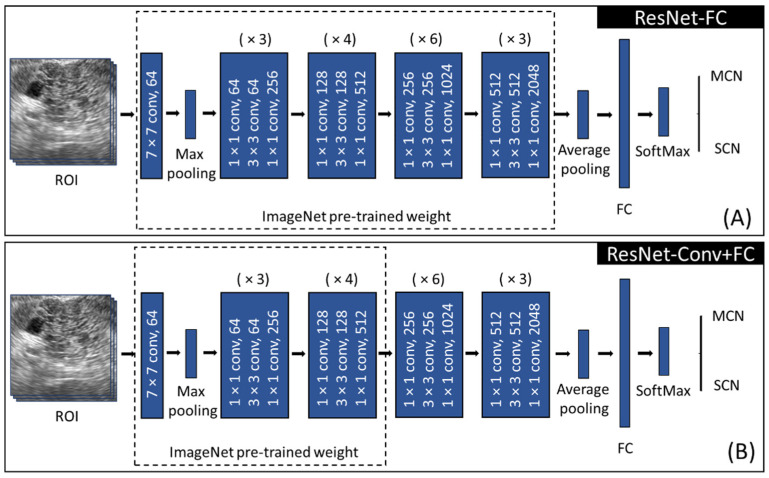
Training approach for (**A**) ResNet-FC, (**B**) ResNet-Conv+FC utilizing ResNet-50. (ROI: region of interest, FC: fully connected layer, MCN: mucinous cystic neoplasm, SCN: serous cystic neoplasm, (×*N*): *N* times block repetition).

The CNN model used in this study was Resnet-50 [24], which consists of four main blocks (Figure 6). Each block contains several convolutional operations as shown in Figure 6. Resnet introduced residual learning by using a skip connection technique to overcome the vanishing and exploding gradient problem during the training of the deep layer.

Owing to the limited training data size, the transfer learning technique was employed instead of training from scratch [21,22,25,26,27]. The idea of transfer learning is to reuse the trained weights learned from a similar task. There are several fine-tuning strategies in transfer learning, including determining whether to freeze or to train the selected layers [27]. The level of fine-tuning in transfer learning can be decided based on the similarity between the pre-training task and the target task and also the characteristics of the data to be utilized. In this study, the weights pre-trained on the ImageNet data, which consists of approximately 15 million labeled high-resolution natural images, were utilized. As the characteristics of EUS data differ from those of ImageNet data, two fine-tuning approaches with transfer learning were employed as shown in Figure 6. First, the fully connected layer (2048 × 2) was trained, followed by a softmax layer (Figure 6A). This approach is denoted by Resnet-FC, which utilizes most of the features from the pre-trained weight. Another fine-tuning approach was implemented by freezing the first three stages in the feature selection part while the remaining stages were trained (Figure 6B), which is denoted by ResNet-Conv+FC. This keeps low level features from the pre-trained weight but extracts high level features and classifiers from the EUS data. Both ResNet-FC and ResNet-Conv+FC were trained using the hyperparameters listed in Table 2. They were trained and tested using an Intel^®^ Core™ i7-9700 CPU @ 3.00GHz processor, a CUDA-enabled Nvidia GTX 1660 Ti graphical processing unit (GPU), and Python 3.8 and PyTorch 1.7.1.

#### 2.2.2. Grad-CAM

Although a deep learning network can be trained to classify the EUS images by differentiating MCN and SCN, it is hard to clarify how the decision was made. In this study, gradient-weighted class activation mapping (Grad-CAM) [22,28] was used to generate a heatmap visualizing the region or feature that played an important role in making decisions from any *k*-th feature in the network.

The Grad-CAM map $L_{Grad-CAM}^{c}$ can be obtained as follows: first, compute $\alpha_{k}^{c}$, i.e., the neuron importance weights, by giving an input image to the network to obtain the score $y^{c}$ of a specific class C, which is either MCN or SCN in this study, as specified in Equation (1). The gradient of all other classes is set to 0, whereas the target class C is set to 1. The gradient is then calculated via the backpropagation that flows to the *k*-th-feature map $A^{k}$ over i (width) and j (height) while Z is the number of pixels in $A^{k}$. Finally, we can obtain the $L_{Grad-CAM}^{c}$ by a weighted combination of $A^{k}$ and $\alpha_{k}^{c}$ followed by a ReLU activation function as shown in Equation (2).

(1) $$\alpha_{k}^{c}=\frac{1}{Z}\underset{i}{\sum }\underset{j}{\sum }\frac{\partial y^{c}}{\partial A_{ij}^{k}},$$

(2) $$L_{Grad-CAM}^{c}=Relu\left(\underset{k}{\sum }\alpha_{k}^{c}A^{k}\right).$$

In this study, Grad-CAM from the last convolution layer was calculated by Equations (1) and (2).

### 2.3. Statistical Analyses

An Anderson–Darling test and a paired t-test were used to compare the accuracy, sensitivity, and specificity between each group of holdout validation, and to compare the metrics between the two fine-tuning approaches as classifiers, respectively. Statistical differences between AUC values were defined using two-sided DeLong tests, with *p*-value < 0.05 as the statistically significant level.

## 3. Results and Evaluation

### 3.1. Deep Learning Network Training

**Figure 7:**
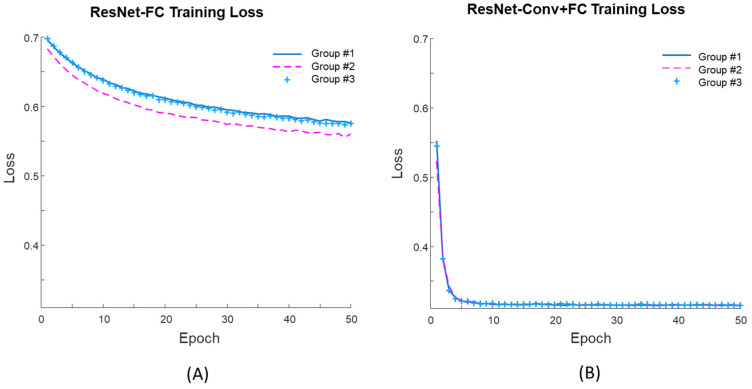
Training loss from three groups for (**A**) ResNet-FC and (**B**) ResNet-Conv+FC.

Figure 7 shows the learning curves during training for both fine-tuning approaches. For the first 50 epochs of the training, the mean training losses of the three groups for the ResNet-FC (Figure 7A) was 0.57 while that of the ResNet-Conv+FC (Figure 7B) was 0.32. For Res-Net-FC, the learning curve reached a stable point after 150 epochs but the loss was still higher than 0.55. Thus, the training learning curve shows that the network can be trained successfully with ResNet-Conv+FC.

### 3.2. Evaluation of the Network Model

**Figure 8:**
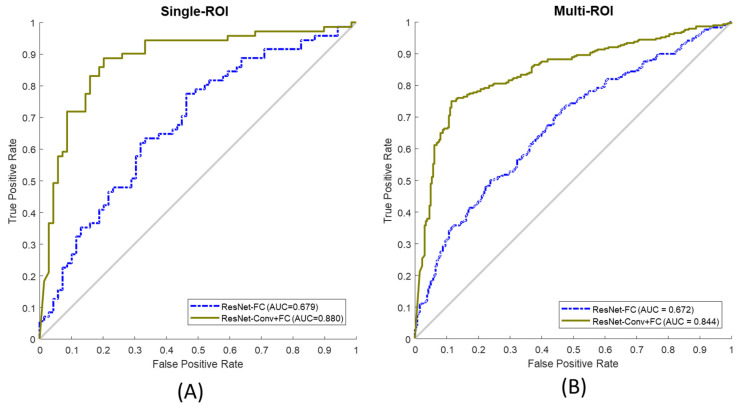
(**A**) ROC curve from single-ROI testing data, (**B**) ROC curve from multi-ROI test data. ResNet-FC and ResNet-Conv+FC were both observed for test data. (ROC: receiver operating characteristic, ROI: region of interest, Resnet-FC: Figure 6A, ResNet-Conv+FC: Figure 6B).

Metrics such as accuracy, sensitivity, specificity, and AUC score taken from the receiver operating characteristic (ROC) were used to evaluate the performance of the network. Figure 8 illustrates the ROC curves of both fine-tuning approaches (solid line: ResNet-Conv+FC and dotted line: ResNet-FC) for single-ROI (Figure 8A) and multi-ROI (Figure 8B). The Anderson–Darling test confirmed that the calculated accuracy, sensitivity, and specificity show normal distribution. Table 3 presents the performance of the two fine-tuning approaches tested on single-ROI and multi-ROI data. Overall, ResNet-Conv+FC achieved higher performance in terms of accuracy (by 20.47%, *p* = 0.0036), sensitivity (by 20.79%, *p* = 0.0045), specificity (by 21.06%, *p* = 0.0046), and AUC score (by 22.73%) compared to ResNet-FC. The result shows that the classification performance of ResNet-Conv+FC is significantly different (*p* < 0.05) and superior compared to that of ResNet-FC.

### 3.3. Gradient Visualization (Grad-CAM)

**Figure 9:**
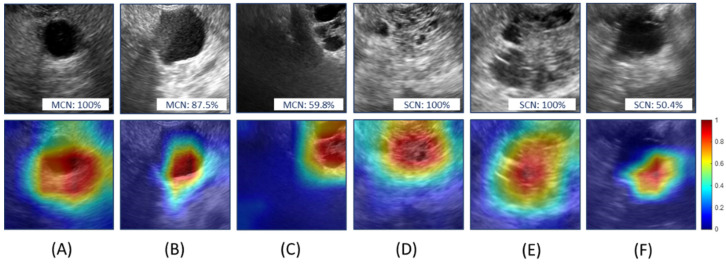
Grad-CAM mapping obtained using ResNet-Conv+FC. The upper row shows the selected ROIs from test images and the lower row shows the corresponding Grad-CAM mappings. (**A**–**C**): mucinous cystic neoplasm and (**D**–**F**): serous cystic neoplasm. Colors from blue to red in Grad-CAM illustrate the degree of decision contribution from various regions.

Figure 9 shows the Grad-CAM mapping obtained from ResNet-Conv+FC. Figure 9A–C are MCN cases (probabilities of the predicted class were 100%, 87.5%, and 59.8%, respectively) while Figure 9D–F are SCN cases (probabilities of the predicted class were 100%, 100%, and 50.4%, respectively). The Grad-CAM mapping emphasizes the regions affecting the decision the most from the network. Colors from blue (low) to red (high) in Grad-CAM illustrate the degree of decision contribution from various regions. It confirms that the network model learned the critical features for the classification. Although Figure 9C,F is correctly predicted, the prediction probabilities were 59.8% and 50.4% for MCN and SCN, respectively. Despite Grad-CAM mapping for Figure 9C,F showing high activation near the cyst region, the shapes of the cyst are hard to differentiate; this affected the decision. Although the network was trained without information about the position of the cysts, it was observed that the decision from the network model was made based on the features learned from the cystic region.

## 4. Discussion

In this study, we implemented a CNN model to differentiate between MCN and SCN using EUS images. Overall accuracy was up to 82.76% and 80.00% from single-ROIs and multi-ROIs, respectively. The classification errors from the implemented network model mostly occurred in the confusing cases. It is known that the diagnosis of the macrocystic SCN using EUS images is difficult even for experienced sonographers [29]. In spite of the fact that the proposed model is not yet able achieve a level of histological diagnosis, the performance of the current study reached the level of experienced endosonographers. By utilizing the features in the images from the CNN model, the network model showed comparable performance to that of Zhang et al. [1]. When their criteria with visual features and additional information including the measured CEA level achieved the accuracy of 82.93%.

There are several approaches to fine-tuning to train the network model [27], but two fine-tuning approaches (ResNet-FC and ResNet-Conv+FC) were shown in this study. Although it is not included in this paper, the full network was trained using initial pre-trained weights and was also compared with various layers selected for training. However, they all showed worse performance than the two fine-tuning approaches selected in this study. Among the two selected fine-tuning approaches (Table 3), the network achieved higher diagnosis performance by training selected top layers (ResNet-Conv+FC) compared to the performance when the full pre-trained weights (Resnet-FC) were utilized.

Owing to the small amount of available data and varying number of samples for each patient, holdout validation was employed to choose training and test data instead of k-fold cross validation. In order to evaluate the reliability of the network, the multi-ROI cases were employed. Varying the size and region of the ROI causes a slight decrease in the overall accuracy. Considering that the EUS images were taken from two different hospitals using various kinds of EUS imaging settings and clinical configurations (e.g., radial and linear echoendoscopes), however, the results in this study shows that our approach can differentiate between MCN and SCN without dependency on the variations of EUS imaging conditions.

Diagnosing various kinds of PCN remains a challenge owing to the lack of datasets and exact criteria for differentiation. Typically, unilocular or multilocular cysts with variable septations on an EUS image can be defined as MCN [30]. When a cyst has peripheral calcifications or mucin content on EUS images, it is also categorized as MCN [30,31]. When a cyst shows micro-cystic (<3 mm) honeycomb structures, it is classified as typical SCN [31]. In addition, a cyst with a central scar can also be diagnosed as SCN [30,31]. The IPMN case is especially complicated as it needs to consider surrounding tissues and has a high chance of developing multiple cysts, which requires several EUS images to make the decision rather than just a single image. Thus, further investigation is necessary to improve the current network model to work with multiple input images to differentiate various kinds of PCN cases. Owing to the scanning condition of the endoscopy, an EUS can have varying distances from the surface of the imaging target and each imaging frame encounters huge variations. Sequential frames in the EUS video can be utilized to overcome this issue. Only two kinds of PCNs were diagnosed in this study because of the limitations of the dataset, but the results in this study indicate the possibility of expanding the CNN model for the differentiation of PCNs from EUS images. Although the performance of the network was limited by the small amount of data in this study, the network will be further improved to differentiate various kinds of PCN cases.

## 5. Conclusions

The implemented network provides reliable performance in differentiating between MCN and SCN in terms of accuracy, sensitivity, and AUC score with verification from Grad-cam mapping. In the future, we will further improve the network to differentiate various kinds of PCN cases and also difficult cases using increased amounts of EUS images and video data for the network training.

## Figures and Tables

**Table 1:** Patient and lesion characteristics. (MCN: mucinous cystic neoplasm, SCN: serous cystic neoplasm).

Gender (Male/Female)	38/71
Mean age, years (range)	60 (19–88)
Average cyst size, mm (range)	27.5 (4–75)
Locularity (monolocular/multicystic)	51/58
Cyst location (head/body/tail)	55/20/37
Histology (MCN/SCN)	18/26
FNA (MCN/SCN)	3/2
Recorded year (range)	2010–2020

**Table 2:** Hyperparameters for the training.

Number of Epochs	50
Batch size	32
Learning rate	1 × 10^−5^
Optimizer	Adam (weight decay 1 × 10^−4^)
Loss function	Cross entropy

**Table 3:** Model performance for single ROI test set and multi-ROI test set (Bold font: best results).

Test Set	Fine-Tune Approach	Accuracy	Sensitivity	Specificity	AUC (95% CI)
Single-ROI	ResNet-FC	62.29%	60.67%	63.30%	0.68 (0.640–0.794)
	ResNet-Conv+FC	**82.76%**	**81.46%**	**84.36%**	**0.88 (0.817–0.930)**
Multi-ROI	ResNet-FC	62.56%	60.35%	64.14%	0.67 (0.631–0.711)
	ResNet-Conv+FC	**80.00%**	**76.06%**	**84.55%**	**0.84 (0.811–0.873)**

## Data Availability

The data are not publicly available due to privacy and ethical concerns.
